# Concordance of bioactive vs. total immunoreactive serum leptin levels in children with severe early onset obesity

**DOI:** 10.1371/journal.pone.0178107

**Published:** 2017-05-23

**Authors:** Juraj Stanik, Jürgen Kratzsch, Kathrin Landgraf, Kathrin Scheuermann, Ulrike Spielau, Ruth Gausche, Daniela Gasperikova, Wieland Kiess, Antje Körner

**Affiliations:** 1 Center for Pediatric Research Leipzig, University Hospital for Children & Adolescents, University of Leipzig, Leipzig, Germany; 2 First Department of Pediatrics, Medical Faculty at the Comenius University, Bratislava, Slovakia; 3 DIABGENE Laboratory, Institute of Experimental Endocrinology, Biomedical Research Center, Slovak Academy of Sciences, Bratislava, Slovakia; 4 Institute of Laboratory Medicine, Clinical Chemistry and Molecular Diagnostics, University of Leipzig, Leipzig, Germany; 5 Integrated Research and Treatment Center Adiposity Diseases, University of Leipzig, Leipzig, Germany; 6 CrescNet, University of Leipzig, Leipzig, Germany; Baylor College of Medicine, UNITED STATES

## Abstract

**Context:**

Leptin secreted from adipose tissue signals peripheral energy status to the brain. Monogenic leptin deficiency results in severe early onset obesity with hyperphagia. Recently, a similar phenotype of inactivating leptin mutations but with preserved immunoreactivity and hence normal circulating immunoreactive leptin has been reported.

**Objective:**

We aimed to evaluate the proportion of bioactive leptin serum levels (compared to immunoreactive leptin) as a biomarker for the screening of leptin gene mutations causing monogenic obesity. Furthermore, we aimed to compare the immunoreactive and bioactive leptin levels associations with parameters of insulin resistance and insulin secretion in obese children and adolescents.

**Patients and methods:**

We measured bioactive and immunoreactive leptin levels by enzyme-linked immunosorbent assays in fasting serum samples of 70 children with severe (BMI SDS >3) non-syndromic obesity with onset <3 years of life from our Leipzig childhood obesity cohort (n = 1204). Sanger sequencing of the leptin gene was performed in probands with proportion of bioactive/immunoreactive leptin <90%.

**Results:**

The mean levels of bioactive and immunoreactive leptin were almost identical (41.1±25.2 vs. 41.1±25.4ng/mL). In three probands with the lowest bioactive leptin proportion (<90%) we did not identify mutations in the leptin gene. Compared to immunoreactive leptin, bioactive leptin showed similar and slightly better statistical associations with indices of insulin resistance in correlation and multivariate analyses.

**Conclusion:**

In our sample selected for severe early onset childhood obesity, we did not identify leptin gene mutations leading to decreased proportion of bioactive leptin.

Nevertheless, the bioactive leptin levels were stronger associated with selected insulin secretion/resistance indices than the immunoreactive leptin levels.

## Introduction

Leptin is a circulating (adipo)cytokine produced mainly by adipose tissue and acts as a satiety signal after binding to its receptor in the hypothalamus [1]. Leptin levels are closely related to, or even reflecting, body fat mass and obesity associated metabolic parameters in both mice and humans [2]. Rodent models of impaired leptin functionality show progressive obesity due to exaggerated food intake [3]. In humans, only rare monogenic cases of leptin deficiency due to the biallelic nonsynonymous mutations of the leptin gene with almost undetectable circulating leptin levels have been identified [4]. These patients had an early onset of severe obesity, hyperphagia and pubertal delay [4]. Very recently, two children with early onset massive obesity due to biallelic missense mutations of the leptin gene leading to formation of leptin with impaired functional activity but preserved immunoreactivity and hence normal leptin levels have been identified [5, 6]. Both, patients with leptin deficiency or impaired leptin bioactivity, could benefit from the treatment with leptin analogues with substantial weight loss and a normal course of puberty [5–7]. It may hence be worthwhile to screen patients with severe early onset obesity for leptin insufficiency and to subject them to pharmacologic treatment.

Nevertheless, the phenotype of patients with deficiency or impaired bioactivity of leptin may not be easy to differentiate from common severe obesity and the condition could be underdiagnosed.

Leptin measurement can be performed with immunoassays that characterize the immunoreactive concentration of the leptin molecule. However, the assessment of immunoreactive leptin plasma levels would not allow detecting leptin gene mutation carriers with impaired leptin bioactivity but preserved immunoreactivity. Alternatively, binding assays may estimate the leptin concentration capable to bind with its soluble receptor and thus specifically determine the concentration of the bioactive protein. Such assays for bioactive leptin are now available [6].

We hypothesize that the ratio of bioactive leptin to immunoreactive leptin could serve as a biomarker for impaired leptin bioactivity due to undetected leptin gene mutations causing monogenic obesity. We, therefore, aimed to use proportion of bioactive leptin serum levels (compared to immunoreactive leptin) as a biomarker for the screening of leptin gene mutations. Furthermore, we hypothesize, that a stronger association with insulin resistance parameters should be seen with bioactive, compared to the immunoreactive leptin levels and we, therefore, compared the immunoreactive and bioactive leptin levels associations with parameters of insulin resistance and insulin secretion in obese children and adolescents.

## Materials and methods

### Study subjects

Probands for the study were selected from the Leipzig childhood obesity cohort (n = 1204, age range 1.0–19.8 years, BMI SDS range 1.88–5.67) [8]. We excluded 36 probands with syndromic forms of obesity. From the remaining individuals, 70 probands with the earliest obesity onset (<3 years), and the highest maximum BMI SDS (>3.0 during the childhood and adolescence) were selected. Clinical features of the 70 selected probands (47 females, 23 males; 46 prepubertal, 11 pubertal, and 13 postpubertal) are shown in Table 1.

**Table 1:** Phenotype characterization of 70 early-onset severely obese children and adolescents selected for the leptin measurements.

	Bioactive leptin proportion >90%	Bioactive leptin proportion <90%	p	Whole group
**n**	67	3		70
**Males/Females**	22/45	1/2	0.986	23/47
**Age (years)**	8.6 ± 4.9	6.5 ± 2.1	0.471	8.5 ± 4.9
**Age of obesity onset (years)**	0.7 ± 0.9	0.2 ± 0.3	0.310	0.7 ± 0.9
**Height (cm)**	136.5 ± 26.3	130.2 ± 15.5	0.685	136.2 ± 25.9
**Height-SDS**	1.1 ± 1.1	1.7 ± 0.8	0.383	1.2 ± 1.1
**Weight (kg)**	67.2 ± 41.6	56.5 ± 12.5	0.659	66.7 ± 40.8
**Weight-SDS**	3.7 ± 0.7	4.1 ± 0.9	0.300	3.7 ± 0.7
**BMI (kg/m**^**2**^**)**	32.2 ± 8.8	33.2 ± 4.7	0.847	32.3 ± 8.6
**BMI-SDS**	3.5 ± 0.5	4.0 ± 0.9	0.163	3.5 ± 0.5
**Maximum BMI-SDS**	3.9 ± 0.8	4.6 ± 0.9	0.135	3.9 ± 0.8
Pubertal category*	0.3 ± 0.5	0.3 ± 0.6	0.985	0.3 ± 0.5
**G**_**0**_ **(mmol/L)**	5.2 ± 0.6	5.2 ± 0.1	0.957	5.2 ± 0.6
**INS**_**0**_ **(pmol/L)**	129.7 ± 143.2	89.2 ± 2.9	0.629	127.9 ± 140.2
**HbA1c (%) (mmol/mol)**	5.4 ± 0.5 (36.0 ± 5.4)	5.5 ± 0.2 (37.0 ± 2.3)	0.747	5.4 ± 0.5 (36 ± 5.3)
**TC (mmol/L)**	4.3 ± 1.0	4.0 ± 1.1	0.627	4.3 ± 1
**HDL (mmol/L)**	1.2 ± 0.3	1.1 ± 0.3	0.596	1.2 ± 0.3
**TG (mmol/L)**	1.3 ± 0.8	0.8 ± 0.04	0.289	1.3 ± 0.7
**Free fatty acids (mmol/L)**	0.8 ± 0.3	0.8 ± 0.3	0.842	0.8 ± 0.2
**Bioactive leptin (ng/mL)**	40.8 ± 25.4	45.8 ± 23.4	0.743	41.1 ± 25.2
**Immunoreactive leptin (ng/mL)**	40.7 ± 25.5	51.9 ± 25.3	0.459	41.1 ± 25.4
**Proportion of bioactive leptin (%)**	101.3 ± 5.5	87.5 ± 3.4	**<0.001**	100.7 ± 6.1
**Soluble leptin receptor**	17.4 ± 7.0	12.4 ± 5.9	0.234	17.2 ± 7.0

Data are expressed as mean ± SD. The significant differences (p<0.05) between probands with proportion of bioactive leptin <90% and those with >90% in a t-test are marked in bold. Abbreviations: BMI—body mass index; SDS—standard deviation score; G_0_—fasting glucose levels, INS_0_—fasting insulin levels; HbA1c –glycosylated hemoglobin A1; TC—immunoreactive cholesterol; TG–triglycerides. Maximum BMI-SDS symbolizes the highest BMI-SDS value obtained during the regular checks by pediatric endocrinologist or general practitioner.

*Pubertal category: 0 = prepubertal, 1 = pubertal, 2 = postpubertal (adolescent).

The body mass index (BMI) and other anthropometric values were standardized referring to sex and age specific national reference data [9]. Pubertal status was evaluated using the Tanner criteria, and divided into three categories prepubertal (P1), pubertal (P2 to P4), and postpubertal (adolescent) (P5).

Written consent was obtained from both parents and from children >12 years. The study was approved by the ethics committee of the University of Leipzig (NCT 02208141).

### Biochemical analyses

Both, immunoreactive and biologically active leptin concentrations were measured from fasted serum samples by enzyme-linked immunosorbent assays using the bioactive leptin kits from Mediagnost (Reutlingen) with a detection limit of 0.01 ng/mL and interassay coefficients of variation (CV) below 6.2% at 7.2 ng/mL or 4.6% at 15.0 ng/mL and the immunoreactive leptin kits (sensitive ELISA, Mediagnost, Reutlingen), with a detection limit of 0.014 ng/mL, and an interassay CVs below 7.5% for samples at 14.9 ng/mL, 6.93 and 2.04 ng/mL. Proportion of bioactive to immunoreactive leptin was evaluated as ratio between both concentrations [10]. Soluble leptin receptor concentrations were measured from frozen serum by enzyme-linked immunosorbent assays using kits from Mediagnost (Reutlingen). The detection limit was <0.01 ng/mL, intra- and inter-assay CVs were below ≤10.9%.

Glucose, lipids, and insulin were measured by standard laboratory protocols in the Institute of Laboratory Medicine of the University of Leipzig, Germany. Both the fasting measures and the oral glucose tolerance (oGTT) test with 75g glucose were performed using a standardized protocol (by WHO) following a 10-hour overnight fast.

### Genetic analyses

We performed genetic analyses of the *LEP* gene in probands with proportion of bioactive leptin <90% (<5^th^ percentile). Genomic DNA was extracted from peripheral leukocytes using standard procedures, and the coding exons and intron/exon boundaries of the *LEP* gene were amplified by polymerase chain reaction (PCR) using previously described primers [4]. PCR products were sequenced using standard methods on an ABI/Hitachi 3500 (Applied Biosystems, Warrington, UK) and were compared with the reference sequence NM_000230.2 using SeqScape software (version 2.1.1; Applied Biosystems, Warrington, UK).

### Assessment of metabolic parameters

We excluded individuals treated with metformin (n = 2). For assessment of insulin secretion and or resistance we selected five fasting and five oGTT derived indices commonly used for characterization of insulin secretion and insulin resistance (Supplement 1): fasting insulin (INS_0_) [11], Homeostatic model assessment—insulin resistance (HOMA-IR) [12], Quantitative insulin sensitivity check index (QUICKI) [13], insulin sensitivity index with free fatty acids (ISI-FFA) [14], C-peptide/fasting glucose ratio (CP/GLU) [15], 120-minute insulin during an oGTT (INS_120_) [11], peak insulin level during an oGTT (INS_MAX_) [16], oral disposition index (oDI) [17], ratio of areas under the curve for insulin and glucose levels during an oGTT (AUC_INS/_AUC_GLU_) [18], and the whole body insulin sensitivity index (WBISI Matsuda) [19]. Where appropriate, glucose and insulin concentrations were transformed from mmol/L and nmol/L to mg/dL and ng/mL using coefficients of 0.05551 for glucose, and 6.945 for insulin, respectively.

### Statistical analyses

Values for the cohort description are given as mean±SD. Non-normally distributed parameters were logarithmically transformed prior to further statistical analyses. Comparison between groups was tested using t-test. Parameters of insulin resistance and bioactive leptin and immunoreactive leptin levels were included to the Pearson´s partial correlations and multiple regression analyses with the stepwise forward model. As co-variables in both analyses we included sex, age, BMI-SDS, pubertal state, and HbA1c (all of them were previously tested in Pearson´s correlations and univariate regression analysis with at least one insulin resistance index with the significance level p<0.2). P values less than 0.05 were considered as statistically significant. Statistical analyses were performed with Statistica 10 software (Dell, Round Rock, US).

## Results

### Bioactive and immunoreactive leptin serum concentrations

**Fig 1:**
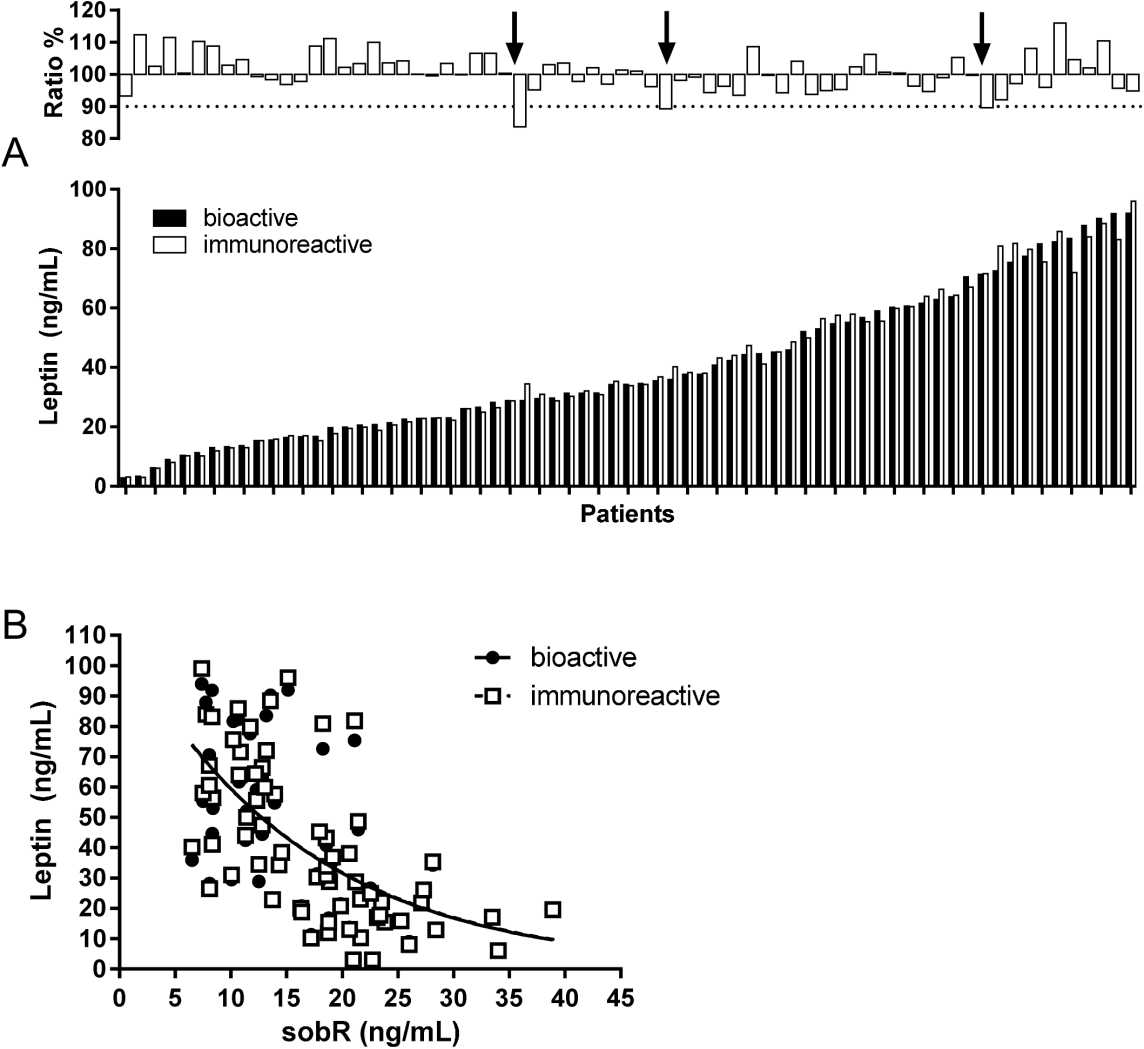
Distribution of bioactive and immunoreactive leptin levels. A–the range of bioactive and immunoreactive leptin for individual patients is shown as indicated (lower part). Ratio between both forms ranged around 100% with few patients lower than 90% (upper part). B–Non-linear regression between bioactive and immunoreactive leptin serum levels with soluble leptin receptor (sobR).

In order to evaluate bioactive leptin as a biomarker for leptin gene mutations and insulin resistance, we have selected 70 probands with early-onset severe obesity. Bioactive leptin serum levels varied from 2.9 to 93.9 ng/mL (mean±SD: 41.1±25.2 ng/mL). Corresponding immunoreactive leptin levels showed variations from 3.0 to 99.1 ng/mL (41.1± 5.4 ng/mL) (Fig 1A). Overall, bioactive and immunoreactive leptin levels were highly concordant with ratios between 83.6% to 116.0% (100.7 ± 6.1%). Both, bioactive and immunoreactive leptin showed an exponential negative correlation to the soluble leptin receptor sobR with similar regression lines (Fig 1B). We did not find any significant differences between females and males in the t-test or partial correlations controlled for age, BMI-SDS and pubertal state for bioactive leptin (43.5 ± 25.5 ng/mL for females and 36.0 ± 24.3 ng/mL for males, p = 0.245 in t-test, and R = 0.00, p = 0.997 in partial correlation, respectively), immunoreactive leptin (43.6 ± 25.9 ng/mL for females and 36.2 ± 24.3 ng/mL for males, p = 0.256 in t-test, and R = 0.012, p = 0.926 in partial correlation, respectively), and proportion of bioactive leptin serum levels compared to immunoreactive leptin (101.0 ± 6.6% for females and 100.2 ± 4.9% ng/mL for males, p = 0.605 in t-test, and R = -0.110, p = 0.386 in partial correlation, respectively).

### Bioactive leptin in monogenic obesity

Since it has been demonstrated, that several leptin gene mutations may cause impairment in bioactivity of leptin [5, 6] we performed *LEP* gene analysis by Sanger sequencing in three probands with the lowest proportion of bioleptin (i.e. 83.6%, 89.3%, and 89.7%, respectively). We did not find mutations in the coding region of the leptin gene.

### Leptin and insulin resistance

**Fig 2:**
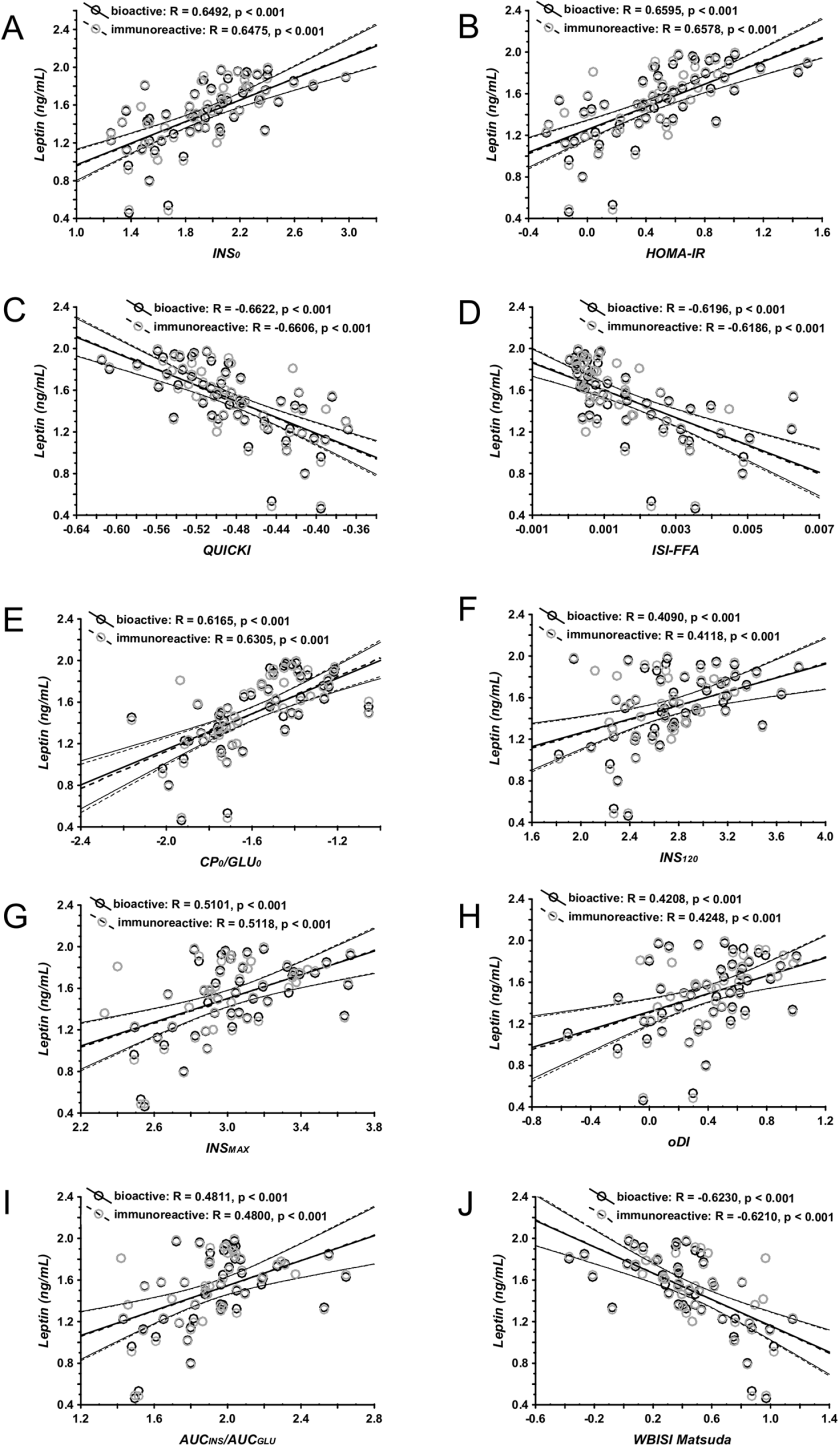
Correlation between bioactive and immunoreactive leptin levels with selected indices of insulin secretion and insulin resistance. A—fasting serum insulin (logINS_0_); B–HOMA-IR; C–QUICKI; D–free fatty acids insulin sensitivity index (ISI-FFA); E–C-peptide and fasting glucose ratio (CP_0_/GLU_0_); F– 120-minute values of insulin during a 75g oral glucose-tolerance test (INS_120_); G–peak insulin levels during a 75g oral glucose-tolerance test (INS_MAX_); H–oral disposition index (oDI); I–ratio of areas under the curve for insulin and glucose levels during a 75g oral glucose-tolerance test (AUC_INS/_AUC_GLU_); J–whole body insulin sensitivity index (WBISI Matsuda). Empty black icons symbolize bioactive leptin, and empty grey icons immunoreactive leptin levels, respectively. In the figure log values of all variables were used.

All selected insulin secretion/resistance indices (Supplement 1) correlated significantly with both, bioactive and immunoreactive leptin levels in Pearson´s correlations (Fig 2). In partial correlations adjusted for sex, age, BMI SDS, pubertal status, and glycosylated hemoglobin HbA1c, bioactive leptin correlated significantly with all of the selected indices; immunoreactive leptin levels correlated significantly with only 8 of 10 indices (80%). Moreover, correlations with bioactive leptin were stronger in 8 of 10 indices (R_bioleptin_>R_leptin_; p_bioleptin_<p_leptin_) (Table 2). Also, in multiple regression analyses, bioactive leptin levels were slightly superior to immunoreactive leptin levels in predicting insulin secretion/resistance indices (Table 3).The further inclusion of the soluble leptin receptor concentrations as co-variable into the multiple regression analyses rather weakened the models (S2 Table).

**Table 2:** Partial correlation between bioactive or immunoreactive leptin levels controlled for sex, age, BMI SDS, pubertal stage, and HbA1c and selected insulin secretion or resistance indices.

	log Bioactive leptin		log Immunoreactive leptin	
Index	R	p	R	p
**log INS**_**0**_	0.311	**0.047**	0.290	0.057
**log HOMA-IR**	0.306	**0.044**	0.293	0.053
**log QUICKI**	-0.326	**0.031**	-0.316	**0.037**
**log ISI-FFA**	-0.356	**0.018**	-0.354	**0.019**
**log CP**_**0**_**/GLU**_**0**_	0.358	**0.017**	0.372	**0.013**
**log INS**_**120**_	0.325	**0.031**	0.328	**0.030**
**log INS**_**max**_	0.415	**0.005**	0.413	**0.005**
**log oDI**	0.331	**0.028**	0.330	**0.029**
**log AUC**_**INS**_**/AUC**_**GLU**_	0.389	**0.009**	0.386	**0.010**
**log WBISI Matsuda**	-0.348	**0.021**	-0.344	**0.022**

The significant correlations (p<0.05) are marked in bold. Abbreviations: fasting serum insulin (logINS_0_); Homeostatic model assessment—insulin resistance—HOMA-IR (log HOMA-IR); Quantitative insulin sensitivity check index—QUICKI (log QUICKI); free fatty acids insulin sensitivity index (log ISI-FFA); C-peptide and fasting glucose ratio (log CP/GLU); 120-minute values of insulin during a 75g oral glucose-tolerance test (logINS_120_); peak insulin levels during a 75g oral glucose-tolerance test (log INS_MAX_); oral disposition index (log oDI); ration of areas under the curve for insulin and glucose levels during a 75g oral glucose-tolerance test (log AUC_INS/_AUC_GLU_); whole body insulin sensitivity index (log WBISI Matsuda).

**Table 3:** Multiple regression analyses for insulin secretion and resistance indices and bioactive and immunoreactive leptin levels.

Step	Parameter	ΔR2	β±SEM	p value
Independent variables for all models: sex, log age, BMI SDS, pubertal status, log HbA1c, log bioactive leptin, and log immunoreactive leptin				** **
Dependent variable: log INS0 (R2 = 0.52; P < 0.001; n = 49)				** **
1	log age	0.437	0.37 ± 0.16	**0.025**
2	log bioactive leptin	0.048	0.3 ± 0.16	0.063
3	log HbA1c	0.023	0.15 ± 0.11	0.159
4	Sex	0.016	0.13 ± 0.11	0.225
Dependent variable: log HOMA-IR (R2 = 0.56; P < 0.001; n = 49)				
1	log age	0.455	0.38 ± 0.15	**0.018**
2	log bioactive leptin	0.052	0.3 ± 0.15	0.052
3	log HbA1c	0.036	0.19 ± 0.1	0.068
4	Sex	0.014	0.12 ± 0.1	0.239
Dependent variable: log QUICKI (R2 = 0.54; P < 0.001; n = 49)				
1	log age	0.449	-0.36 ± 0.16	**0.027**
2	log bioactive leptin	0.059	-0.34 ± 0.16	**0.036**
3	log HbA1c	0.022	-0.15 ± 0.1	0.157
4	Sex	0.012	-0.12 ± 0.11	0.281
Dependent variable: log ISI-FFA (R2 = 0.49; P < 0.001; n = 49)				
1	log bioactive leptin	0.426	-0.44 ± 0.16	**0.009**
2	log age	0.037	-0.49 ± 0.23	**0.042**
3	pubertal status	0.015	0.29 ± 0.21	0.169
4	Sex	0.016	-0.14 ± 0.12	0.24
Dependent variable: log CP_0_/GLU_0_ (R2 = 0.45; P < 0.001; n = 49)				
1	log immunoreactive leptin	0.387	0.54 ± 0.12	**<0.001**
2	log HbA1c	0.048	0.25 ± 0.12	**0.039**
3	BMI SDS	0.013	0.12 ± 0.11	0.312
Dependent variable: log INS120 (R2 = 0.23; P = 0.008; n = 49)				
1	log immunoreactive leptin	0.127	0.33 ± 0.14	**0.021**
2	log HbA1c	0.07	0.24 ± 0.14	0.085
3	BMI SDS	0.031	-0.18 ± 0.14	0.183
Dependent variable: log INSMAX (R2 = 0.27; P < 0.001; n = 49)				
1	log bioactive leptin	0.218	0.51 ± 0.13	**<0.001**
2	BMI SDS	0.054	-0.24 ± 0.13	0.072
Dependent variable: log oDI (R2 = 0.20; P = 0.006; n = 49)				
1	log bioactive leptin	0.176	0.45 ± 0.13	**0.002**
2	BMI SDS	0.021	-0.15 ± 0.13	0.274
Dependent variable: log AUCINS/AUCGLU (R2 = 0.28; P < 0.001; n = 49)				
1	log bioactive leptin	0.232	0.52 ± 0.13	**<0.001**
2	BMI SDS	0.046	-0.22 ± 0.13	0.092
Dependent variable: log WBISI Matsuda (R2 = 0.46; P < 0.001; n = 49)				
1	log bioactive leptin	0.355	-0.55 ± 0.12	**<0.001**
2	log HbA1c	0.055	-0.21 ± 0.12	0.075
3	BMI SDS	0.025	0.15 ± 0.11	0.192
4	Sex	0.021	-0.15 ± 0.11	0.203

The significant correlations (p<0.05) are marked in bold. Abbreviations: fasting serum insulin (logINS_0_); HOMA-IR (log HOMA-IR); QUICKI (log QUICKI); free fatty acids insulin sensitivity index (log ISI-FFA); C-peptide and fasting glucose ratio (log CP/GLU); 120-minute values of insulin during a 75g oral glucose-tolerance test (logINS_120_); peak insulin levels during a 75g oral glucose-tolerance test (log INS_MAX_); oral disposition index (log oDI); ration of areas under the curve for insulin and glucose levels during a 75g oral glucose-tolerance test (log AUC_INS/_AUC_GLU_); whole body insulin sensitivity index (log WBISI Matsuda).

## Discussion

### Leptin as a potential biomarker for leptin gene mutations causing monogenic obesity

We have tested the hypothesis of using the ratio of bioactive leptin to immunoreactive leptin as a biomarker for impaired leptin bioactivity due to leptin gene mutations. In our subset selected for highest expected likelihood for leptin gene mutations we did, however, not identify any patients harboring impaired leptin bioactivity.

Functionally relevant leptin gene mutations are rare. Although a phenotype of massive obesity due to hyperphagia with early onset (and potentially slim parents) may be suggestive, the clinical course may not be so specific to distinguish the carriers from other individuals with severe forms of common obesity. Hence, a biomarker measurable by rather simple means of immunoassay would be beneficial compared to genetic analyses as there appears to be a benefit for the mutation carriers with monogenic obesity from the leptin replacement [20]. However, using this approach, we were not able to identify any leptin gene mutation carriers among three probands with an early-onset severe obesity and the lowest bioactive leptin proportion (<90%). Reasons for a negative result could be explained by several issues, most likely due to the very low frequency of pathogenic leptin gene mutations with only few patients from mostly consanguineous families having been identified so far [1]. So far, about a dozen functionally relevant mutations leading have been described [21]. Of the 64 functional variants listed for the *LEP* gene (ENSEMBL ENSG00000174697) causing alterations in splice sites, stop codon gain, frameshift or missense mutations, only three had a minor allele frequency between 0.001 and 0.005 and one had a minor allele frequency of 0.28. This variant (rs17151919) was, however, only prevalent in African or American populations and not in European Caucasian populations. All other listed variants had a frequency <0.001 or frequency couldn´t even be estimated, which further underscores that monogenic leptin deficiency is a very rare condition. Nevertheless, all previously described leptin gene mutation carriers with leptin deficiency or leptin bioinactivity had an extreme obesity phenotype with an early onset severe obesity. Therefore, we premise that increasing the number of individuals included to our study with children and adolescents with only a lighter obesity phenotype would not increase the probability of identifying a causal leptin gene mutation.

In the described patients with impaired leptin bioactivity but preserved immunoreactivity due to homozygous leptin gene mutations [5, 6] the proportion of bioactive leptin serum levels compared to immunoreactive leptin was much lower. Also, their family relatives carrying heterozygous mutations of the leptin gene had the proportion of bioactive leptin <55% [10]. Nevertheless, in our cohort we did not identify any patient with such a low proportion of bioactive from immunoreative leptin levels.

Also the selection criteria for leptin serum measurements and leptin gene analysis could be an issue. Theoretically, for mutations that affect only bioactivity but not immunoreactivity the likelihood of only partial loss of function is much higher compared to the mutations that reduce the immunoreactivity of the leptin molecule. Hence, screening a larger and less selected sample of obese children is worthwhile considering. Nevertheless, syndromic or monogenic obesity most often presents with early onset massive obesity, which were the criteria for prioritizing our patient selection. The assay conditions also need to be considered to avoid false conspicuous results. There are two preconditions for the measurement of bioactive leptin and the correct interpretation of the proportion of bioactive from immunoreative leptin levels; first, the calibration of both assays has to be done with the same leptin reference preparation, second; the matrix (dilution of the sample and sample diluent) of the used serum samples should be largely identical to avoid interfering effects in the respective assay (data not shown).

Alterations in post-translational modification might also have a physiological and pathophysiological relevance, potentially driven by variants. This may to some extent explain the huge range of leptin levels in obese individuals. Nevertheless, the very close correlation of the two leptin measurements strongly argue against such modification variants. Some of the leptin levels in our cohort of obese individuals were low, but similar findings have been already described in other studies [10]. There were no apparent outliers of bioactive or immunoreactive leptin levels in our study (S1 Fig) compared to majority of heterozygous or homozygous leptin gene mutation carriers who have much lower leptin levels than the non-carriers [4, 22]. Moreover, bioactive to immunoreactive leptin ratio was not lower compared to other individuals in our cohort unlike the work of Wabitsch et al on leptin gene mutation carriers [10]. Low leptin levels in our cohort also correspond to low fasting insulin levels (S1 Fig).

Limitation of our study is that mutations in the promoter region of the leptin gene were not sequenced in our study. Nevertheless, in this case we would expect similarly decreased levels of both, bioactive and immunoreactive leptin levels and hence no change in the proportion. Another limitation is that we did not include the information on the adipose tissue mass. Adipose tissue mass correlates with the leptin levels and could explain some of the leptin levels variability [23]. Finally, leptin levels, and the proportion of bioactive leptin could be influenced also by other non-genetic factors.

In summary, as no leptin mutation carriers in our study were identified, further studies are needed to evaluate the effectiveness of bioactive leptin proportion as a biomarker for the leptin gene mutations.

### Leptin and insulin secretion/resistance indices

The association of leptin plasma levels with obesity is well known [2]. Nevertheless, in children and adolescents this association is strongly influenced by growth and pubertal development [24]. A leptin resistance with increased leptin levels in blood and decreased sensitivity to leptin in the hypothalamus in the obese individuals has been proposed, which may also confer the association of leptin with insulin resistance parameters [2]. Till recently assays for immunoreactive leptin levels measurement were available only. This is the first study with the aim to compare the associations of bioactive and immunoreactive leptin levels with selected parameters of insulin secretion and insulin resistance. We have shown that bioactive and immunoreactive leptin levels correlated significantly (Fig 1). As expected, stronger associations with the selected indices of insulin resistance and secretion in both partial correlations, and multiple regression models were observed for bioactive leptin compared to immunoreactive leptin levels in the majority of the indices, although admittedly the differences are small. This result could suggest using bioactive leptin instead of immunoreactive leptin in both clinical and experimental studies, respectively. Moreover, we have shown that soluble leptin receptor serum levels may give additional information as independent predictor of insulin resistance in 7 of 10 selected indices.

## Conclusion

In our sample selected for severe early onset childhood obesity, we did not identify leptin gene mutations leading to decreased proportion of bioactive leptin. Generally there was a high concordance between bioactive and immunoreactive leptin levels. Nevertheless, we have shown that the bioactive leptin levels were strongly associated with selected insulin secretion and insulin resistance indices superior to immunoreactive leptin levels.

## Supporting information

S1 Fig Association of bioactive and immunoreactive leptin levels with fasting serum insulin. Empty black icons symbolize bioactive leptin, and empty grey icons immunoreactive leptin levels, respectively.
(DOCX)

S1 Table Selected insulin secretion and insulin resistance indices. Abbreviations: FFA–free fatty acids, CP–C-peptide, G_mean_−mean glucose levels during an oGTT, INSmean−mean insulin levels during an oGTT.
(DOCX)

S2 Table Multiple regression analyses for insulin secretion and resistance indices and bioactive, whole leptin levels, and soluble leptin receptor levels. The significant correlations (p<0.05) are marked in bold. Abbreviations: fasting serum insulin (logINS_0_); HOMA-IR (log HOMA-IR); QUICKI (log QUICKI); free fatty acids insulin sensitivity index (log ISI-FFA); C-peptide and fasting glucose ratio (log CP/GLU); 120-minute values of insulin during a 75g oral glucose-tolerance test (logINS_120_); peak insulin levels during a 75g oral glucose-tolerance test (log INS_MAX_); oral disposition index (log oDI); ration of areas under the curve for insulin and glucose levels during a 75g oral glucose-tolerance test (log AUC_INS/_AUC_GLU_); whole body insulin sensitivity index (log WBISI Matsuda).
(DOCX)
