# Supplementary material for: Concordance of bioactive vs. total immunoreactive serum leptin levels in children with severe early onset obesity
Source: PLoS One. 2017 May 23;12(5):e0178107. doi: 10.1371/journal.pone.0178107 (PMC5441582; doi:10.1371/journal.pone.0178107)
Supplement: S1 Fig — Empty black icons symbolize bioactive leptin, and empty grey icons immunoreactive leptin levels, respectively. (DOCX) [file pone.0178107.s001.docx]

**S1 Fig. Association of bioactive and immunoreactive leptin levels with fasting serum insulin.** Empty black icons symbolize bioactive leptin , and empty grey icons immunoreactive leptin levels, respectively.
