# Supplementary material for: Concordance of bioactive vs. total immunoreactive serum leptin levels in children with severe early onset obesity
Source: PLoS One. 2017 May 23;12(5):e0178107. doi: 10.1371/journal.pone.0178107 (PMC5441582; doi:10.1371/journal.pone.0178107)
Supplement: S1 Table — Abbreviations: FFA–free fatty acids, CP–C-peptide, Gmean−mean glucose levels during an oGTT, INSmean−mean insulin levels during an oGTT. (DOCX) [file pone.0178107.s002.docx]

**S1 Table. Selected insulin secretion and insulin resistance indices.**

| **Index** | **Description** | **Calculation** | **Units** | **Ref.** | **Value** | **Range** |
| --- | --- | --- | --- | --- | --- | --- |
| **INS_0_** | Fasting serum insulin | INS_0_ | INS (pmol/l) | [11] | 124.97 ± 140.41 | 17.9 - 956.4 |
| **HOMA-IR** | Homeostatic model assessment - insulin resistance | G_0_*INS_0_/22.5 | G (mmol/L); INS (μU/mL) | [12] | 4.36 ± 5.32 | 0.53 - 31.89 |
| **QUICKI** | Quantitative insulin sensitivity check index | 1/[logINS_0_+logG_0_] | G (mmol/L); INS (μU/mL) | [13] | 0.33 ± 0.04 | 0.24 - 0.43 |
| **ISI-FFA** | Insulin sensitivity index with free fatty acids | 2/(INS_0_*FFA+1) | INS (μU/mL); FFA (mmol/L) | [14] | 1.004 ± 0.004 | 1 - 1.01 |
| **CP/G_0_** | C-peptide/fasting glucose ratio | CP/G_0_ | G (mg/dL); CP (ng/mL) | [15] | 0.03 ± 0.02 | 0.01 - 0.09 |
| **INS_120_** | 120-minute insulin during an oGTT | INS_120_ | INS (pmol/l) | [11] | 868.95 ± 1023.05 | 66.2 - 6070 |
| **INS_max_** | Peak insulin level during an oGTT | INS_max_ | INS (pmol/l) | [16] | 1330.98 ± 1039.72 | 214.7 - 4684 |
| **oDI** | Oral disposition index | (INS_30_-INS_0_)/(G_30_-G_0_)/INS_0_ | G (mg/dL); INS (μU/mL) | [17] | 3.22 ± 2.38 | 0.28 - 10.08 |
| **AUC_INS_/ AUC_GLU_** | Ratio of areas under the curve for insulin and glucose levels during an oGTT | AUC_INS_/AUC_GLU_ | G (mmol/L); INS (pmol/L) | [18] | 103.1 ± 81.22 | 26.5 - 444.05 |
| **WBISI Matsuda** | Whole body insulin sensitivity index | 10000/SQRT  ((G_0_*INS_0_)*  (G_mean_*INS_mean_)) | G (mg/dL); INS (μU/mL) | [19] | 3.89 ± 3.02 | 0.43 - 14.31 |

Abbreviations: FFA – free fatty acids, CP – C-peptide, G_mean_ – mean glucose levels during an oGTT, INS_mean_ – mean insulin levels during an oGTT
