# Supplementary material for: Concordance of bioactive vs. total immunoreactive serum leptin levels in children with severe early onset obesity
Source: PLoS One. 2017 May 23;12(5):e0178107. doi: 10.1371/journal.pone.0178107 (PMC5441582; doi:10.1371/journal.pone.0178107)
Supplement: S2 Table — The significant correlations (p<0.05) are marked in bold. Abbreviations: fasting serum insulin (logINS0); HOMA-IR (log HOMA-IR); QUICKI (log QUICKI); free fatty acids insulin sensitivity index (log ISI-FFA); C-peptide and fasting glucose ratio (log CP/GLU); 120-minute values of insulin during a 75g oral glucose-tolerance test (logINS120); peak insulin levels during a 75g oral glucose-tolerance test (log INSMAX); oral disposition index (log oDI); ration of areas under the curve for insulin and glucose levels during a 75g oral glucose-tolerance test (log AUCINS/AUCGLU); whole body insulin sensitivity index (log WBISI Matsuda). (DOCX) [file pone.0178107.s003.docx]

**S2 Table.** **Multiple regression analyses for insulin secretion and resistance indices and bioactive, whole leptin levels, and soluble leptin receptor levels.**

| **Step** | **Parameter** | **ΔR^2^** | **β±SEM** | **p value** |
| --- | --- | --- | --- | --- |
| **Independent variables for all models: sex, log age, BMI SDS, pubertal status, log HbA1c, log bioleptin, log whole leptin, and log soluble leptin receptor** |  |  |  |  |
| **Dependent variable: log INS_0_ (R^2^ = 0.56; P < 0.001; n = 49)** |  |  |  |  |
| 1 | log age | 0.437 | 0.26 ± 0.17 | **0.131** |
| 2 | log leptin receptor | 0.053 | -0.24 ± 0.14 | 0.092 |
| 3 | log bioleptin | 0.028 | 0.24 ± 0.16 | 0.148 |
| 4 | log HbA1c | 0.021 | 0.14 ± 0.1 | 0.173 |
| 5 | sex | 0.017 | 0.14 ± 0.11 | 0.206 |
| **Dependent variable: log HOMA-IR (R^2^ = 0.60; P < 0.001; n = 49)** |  |  |  |  |
| 1 | log age | 0.455 | 0.25 ± 0.16 | 0.126 |
| 2 | log leptin receptor | 0.066 | -0.28 ± 0.13 | **0.045** |
| 3 | log HbA1c | 0.041 | 0.18 ± 0.1 | 0.072 |
| 4 | log bioleptin | 0.02 | 0.23 ± 0.15 | 0.14 |
| 5 | sex | 0.015 | 0.13 ± 0.1 | 0.212 |
| **Dependent variable: log QUICKI (R^2^ = 0.59; P < 0.001; n = 49)** |  |  |  |  |
| 1 | log age | 0.449 | -0.23 ± 0.16 | 0.169 |
| 2 | log leptin receptor | 0.07 | 0.29 ± 0.14 | **0.042** |
| 3 | log bioleptin | 0.033 | -0.26 ± 0.15 | 0.102 |
| 4 | log HbA1c | 0.02 | -0.14 ± 0.1 | 0.168 |
| 5 | sex | 0.013 | -0.12 ± 0.1 | 0.251 |
| **Dependent variable: log ISI-FFA (R^2^ = 0.51; P < 0.001; n = 49)** |  |  |  |  |
| 1 | log bioleptin | 0.426 | -0.42 ± 0.13 | **0.003** |
| 2 | log leptin receptor | 0.071 | 0.33 ± 0.13 | **0.017** |
| 3 | sex | 0.013 | -0.11 ± 0.11 | 0.289 |
| **Dependent variable: log CP/GLU_0_ (R^2^ = 0.54; P < 0.001; n = 49)** |  |  |  |  |
| 1 | log whole leptin | 0.387 | 2.1 ± 1.43 | 0.15 |
| 2 | log leptin receptor | 0.076 | -0.39 ± 0.13 | **0.006** |
| 3 | log bioleptin | 0.044 | 0.22 ± 0.11 | 0.053 |
| 4 | log HbA1c | 0.022 | -1.8 ± 1.44 | 0.217 |
| 5 | lBMI SDS | 0.016 | 0.13 ± 0.11 | 0.226 |
| **Dependent variable: log INS_120_ (R^2^ = 0.21; P = 0.004; n = 49)** |  |  |  |  |
| 1 | log leptin receptor | 0.133 | -0.31 ± 0.13 | **0.023** |
| 2 | log HbA1c | 0.08 | 0.29 ± 0.13 | **0.035** |
| **Dependent variable: log INS_MAX_ (R^2^ = 0.32; P = 0.001; n = 49)** |  |  |  |  |
| 1 | log bioleptin | 0.218 | 0.57 ± 0.2 | **0.007** |
| 2 | BMI SDS | 0.054 | -0.27 ± 0.13 | **0.048** |
| 3 | log leptin receptor | 0.019 | -0.27 ± 0.17 | 0.118 |
| 4 | log age | 0.033 | -0.31 ± 0.21 | 0.148 |
| **Dependent variable: log oDI (R^2^ = 0.20; P = 0.006; n = 49)** |  |  |  |  |
| 1 | log bioleptin | 0.176 | 0.45 ± 0.13 | **0.002** |
| 2 | BMI SDS | 0.021 | -0.15 ± 0.13 | 0.274 |
| **Dependent variable: log AUC_INS_/AUC_GLU_ (R^2^ = 0.32; P = 0.002; n = 49)** |  |  |  |  |
| 1 | log bioleptin | 0.232 | 0.52 ± 0.2 | **0.014** |
| 2 | BMI SDS | 0.046 | -0.23 ± 0.13 | 0.082 |
| 3 | log leptin receptor | 0.025 | -0.27 ± 0.17 | 0.127 |
| 4 | log age | 0.017 | -0.22 ± 0.21 | 0.304 |
| **Dependent variable: log WBISI Matsuda (R^2^ = 0.51; P < 0.001; n = 49)** |  |  |  |  |
| 1 | log bioleptin | 0.355 | -0.36 ± 0.14 | **0.016** |
| 2 | log leptin receptor | 0.072 | 0.3 ± 0.14 | **0.034** |
| 3 | log HbA1c | 0.049 | -0.21 ± 0.11 | 0.072 |
| 4 | sex | 0.019 | -0.13 ± 0.11 | 0.231 |
| 5 | BMI SDS | 0.015 | 0.13 ± 0.11 | 0.262 |

The significant correlations (p<0.05) are marked in bold. Abbreviations: fasting serum insulin (logINS_0_); HOMA-IR (log HOMA-IR); QUICKI (log QUICKI); free fatty acids insulin sensitivity index (log ISI-FFA); C-peptide and fasting glucose ratio (log CP/GLU); 120-minute values of insulin during a 75g oral glucose-tolerance test (logINS_120_); peak insulin levels during a 75g oral glucose-tolerance test (log INS_MAX_); oral disposition index (log oDI); ration of areas under the curve for insulin and glucose levels during a 75g oral glucose-tolerance test (log AUC_INS/_AUC_GLU_); whole body insulin sensitivity index (log WBISI Matsuda).
